# Urine from Sexually Mature Intact Male Mice Contributes to Increased Cardiovascular Responses during Free-Roaming and Restrained Conditions

**DOI:** 10.5402/2012/185461

**Published:** 2012-02-09

**Authors:** Dexter L. Lee, Justin L. Wilson

**Affiliations:** Department of Physiology and Biophysics, College of Medicine, Howard University, 520 W Street NW, Washington, DC 20059, USA

## Abstract

Pheromones in the urine regulate aggression of male mice and castrated males produce less of these pheromones. We tested the hypothesis that pheromones in the urine of sexually mature-intact (SMI) males placed in the cage bedding of an individually housed male mouse or in a mouse restrainer would contribute to a significant increase in mean arterial pressure (MAP), heart rate (HR), and activity. Sexually mature male C57BL/6 mice were implanted with a biotelemetry transmitter to measure MAP, HR, and activity. Urine (200 *μ*L) from SMI mice placed in the cages of singularly housed male mice caused significant changes above baseline values for MAP (21 ± 4 mmHg), HR (145 ± 25 bpm), and activity (9 ± 2 counts) when compared to urine from castrated mice-induced MAP (11 ± 3 mmHg), HR (70 ± 15 bpm), and activity (5 ± 1 counts). Pretreatment with terazosin significantly reduced the change in MAP (9 ± 3 mmHg), heart rate (90 ± 15 bpm), and activity (4 ± 2 counts) responses to urine from SMI males. Saline did not significantly increase MAP, HR, or activity in any group. During restraint, urine from SMI mice caused a significant change in MAP (5 ± 0.4 mmHg) and HR (17 ± 1 bpm); urine from castrated mice did not cause a significant increase in MAP and HR. Our results demonstrate that a significant increase in MAP, HR, and activity occurs when male mice are exposed to urine pheromones from SMI males. In summary, pheromones in the urine of SMI male excreted in the cage bedding and mouse restrainers contribute to a significant increase in cardiovascular responses in the absence of direct physical contact with a different male mouse or animal handler.

## 1. Introduction

 Male mice urine pheromones are compounds that are emitted and detected by members of the same species as chemical signals to regulate social behaviors such as aggression [1, 2]. 

Among the important chemical signals described for this species is an androgen-dependent pheromone that provokes fighting and attacks among adult males [3]. The use of these chemical signals can linger on long after the “sender” has left the cage, and communication works as well in total darkness as in broad daylight [4]. Male urine is attractive for females and repellent for other males [5]. While urine from intact females decreases aggression in male mice, the urine of testosterone-treated females is effective in inducing male aggression. Whole urine from sexually mature intact males is sufficient to promote aggression when swabbed on the backs of castrated animals [1]. Castrated males produce reduced quantities of the aggression pheromones and fail to stimulate aggressive behavior from recipient males [6]. Although male mouse pheromones regulate aggression, the contributions of pheromones to cardiovascular responses such as mean arterial pressure and heart rate have not been quantified.

The handling of male mice during cardiovascular research may involve placing male mice in restrainers to measure blood pressure via tail cuff or in a cage previously occupied by a male mouse during telemetry measurements. Such maneuvers may expose male mice to pheromones released by a different male mouse. Previous results show that cage-switch stress, a model of psychosocial stress, causes an increase in the hypertensive response that is initially *α*_1_-receptor dependent and the later phase of the response being *β*_1_-receptor dependent [7]. A different study determined that this model of psychosocial stress appears to be specific for blood pressure rather than to a global impairment in the response to stress [8]. Although the previous studies involved the handling of male mice, the contribution of male urine pheromones to mean arterial pressure, heart rate, and activity was not specifically evaluated.

The magnitude of the cardiovascular responses that are elicited during the detection of pheromones in the urine of SMI male mice is largely unknown. In the present study, we utilize two distinct settings that are commonly used in the field of hypertension to determine the influence of male mice pheromones on mean arterial pressure (MAP), heart rate (HR), and locomotor activity. A distinct advantage of the current study is that radiotelemetry is used to determine the contribution of male mice pheromones to the cardiovascular responses in male mice roaming free in the cage and without direct physical contact of an animal handler or a different male mouse. A mouse restrainer was also used to determine the contribution of male mice pheromones on MAP and HR. The aim of the study is to utilize radiotelemetry and isolate the effects of male pheromones from sexually mature male mice from potentially confounding effects of increased stress on blood pressure. Therefore, the goal of this study is to determine the magnitude of the cardiovascular responses in male mice to the detection of pheromones in the urine of SMI male mice.

## 2. Methods

### 2.1. Animals

 Procedures involving animals were approved by the Institutional Animal Care and Use Committee at Howard University and complied fully with those approved by the American Veterinary Medical Association Panel of Euthanasia. All mean arterial pressure, heart rate, and locomotor activity data were obtained via radiotelemetry. A total of 34 sexually mature male mice were implanted with a blood pressure transmitter during this study. Surgery was performed on male C57BL/6J (Jackson Laboratories C57BL/6J 000664) mice (10 weeks of age) to implant blood pressure transmitters (Data Sciences, PA-C10). Briefly, the mice were anesthetized with isoflurane at 1.5–2% in a stream of 100% oxygen mixed with room air. A Data Sciences transmitter catheter was inserted into the left carotid artery via a 10 mm incision on the ventral neck region over the trachea, and the transmitter body was routed to a subcutaneous pocket in the mid-scapular region. Lidocaine (1% in physiological saline solution) and Pen G (10 mg/kg) was given to infiltrate the incision before skin closure. The incision was closed with sterile, 6-0 Ethichon Opthalmic suture. After recovery, mice were housed in individual cages and monitored daily in the laboratory animal facility with a 12 : 12-hour dark-light cycle and provided with standard laboratory chow and water ad libitum. Recording of mean arterial pressure, heart rate, and locomotor activity was not begun until normal circadian rhythm was reestablished (7 days after surgery). Baseline mean arterial pressure (MAP) was measured 19 h/day, from 1500 to 1000 h the next day. The experiments were conducted at 1000 h. During the experiment, MAP, heart rate, and locomotor activity data were collected at 500 Hz for 5 s/min. MAP, HR, and locomotor activity are expressed as a change in millimeters of Hg, beats per minute, and counts, respectively.

#### 2.1.1. Free-Roaming Experiments

To determine the contribution of male mice pheromones to the cardiovascular responses, a six-inch disposable polyethylene transfer pipet was used to place urine (200 *μ*L) from sexually mature-intact (SMI) males, or castrated mice or saline in the resident cage bedding of an individually housed free-roaming male mouse. Plastic filter tops were used to cover the individual cages that housed a single male mouse and also reduce the influence of pheromones during additional experimental conditions. The plastic filter tops were removed from the top of the cages in order to place the fluid in the cage bedding. The metal grids containing the water bottle and animal chow were not lifted or disturbed during the placement of fluid in the cage bedding. The plastic filter tops were replaced once the fluid was placed in the cage bedding.

 We experimented with larger volumes of urine and saline during experimental and control conditions, respectively (i.e., 500–1000 *μ*L) and determined that the larger volumes of saline also contributed significantly to the cardiovascular responses (i.e., significant changes in mean arterial pressure, heart rate and locomotor activity). Although larger volumes (i.e., 1000 *μ*L) of SMI male urine contributed to significant increases in the cardiovascular responses when compared to the saline-induced cardiovascular responses, the larger volumes (i.e, 1000 *μ*L) of saline also alerted the mice and contributed to a significant increase in mean arterial pressure, heart rate, and locomotor activity from baseline averages. Therefore, we wanted to differentiate between the effect of a large volume of a solution and the effects of pheromones on the cardiovascular responses. From preliminary experiments, it was determined that 200 *μ*L of saline did not contribute to a significant increase in mean arterial pressure, heart rate, or locomotor activity from baseline conditions. Therefore, two hundred microliters were used as the control and experimental volumes.

Terazosin (*α*_1_-receptor antagonist) was given via an intraperitoneal injection (10 mg/kg) 3 h before urine or saline applications. Mice were allowed to recovery for the first hour after the terazosin injection. The subsequent two hours were used to establish terazosin-treated baseline MAP, heart rate (HR), and locomotor activity for free-roaming experiments.

#### 2.1.2. Restrainer Experiments

During the restrainer studies, a single mouse was placed in a mouse restrainer (Kent Scientific) for 25–30 minutes to collect baseline mean arterial pressure and heart rate data. After the baseline data collection, 200 *μ*L of urine from SMI or castrated males or saline was placed in the mouse restrainer. The solution was placed inside the open end of the restrainer and did not touch the mouse at any time during the experiment. Although restraint increased baseline blood pressure and heart rate, we wanted to determine if pheromones in the urine of sexually mature intact males potentiated the blood pressure response during restraint stress. To better determine the effects of the pheromones during restraint stress, mice were not introduced to urine from SMI or castrated males or saline if there were signs of excessive movement in the restrainer. During the terazosin experiments, animals were placed in restrainers one hour after the terazosin injection to establish baseline MAP and HR data.

**Figure 1:**
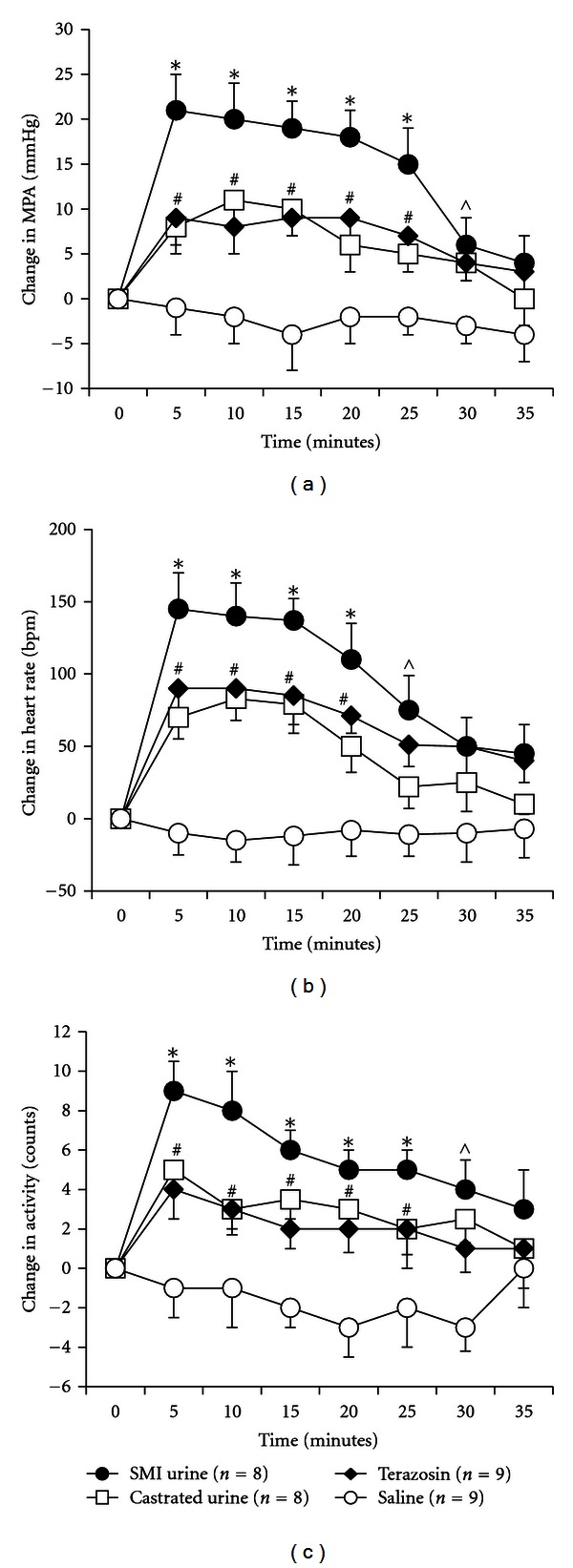
The changes in the hypertensive (a), heart rate (b), and locomotor activity (c) responses of male mice to urine from sexually mature intact (SMI), urine from castrated mice, urine from SMI with terazosin pretreatment, and physiological saline. *Indicates a significant difference in the responses to SMI when compared to all groups. ^#^Indicates a significant difference in the responses between the saline treated group when compared to urine from castrated mice and SMI + terazosin pretreated groups. ^∧^Indicates a significant difference in the responses between saline treated group when compared to SMI Urine, urine from castrated mice, and SMI + terazosin pretreatment (*P* < 0.05).

**Figure 2:**
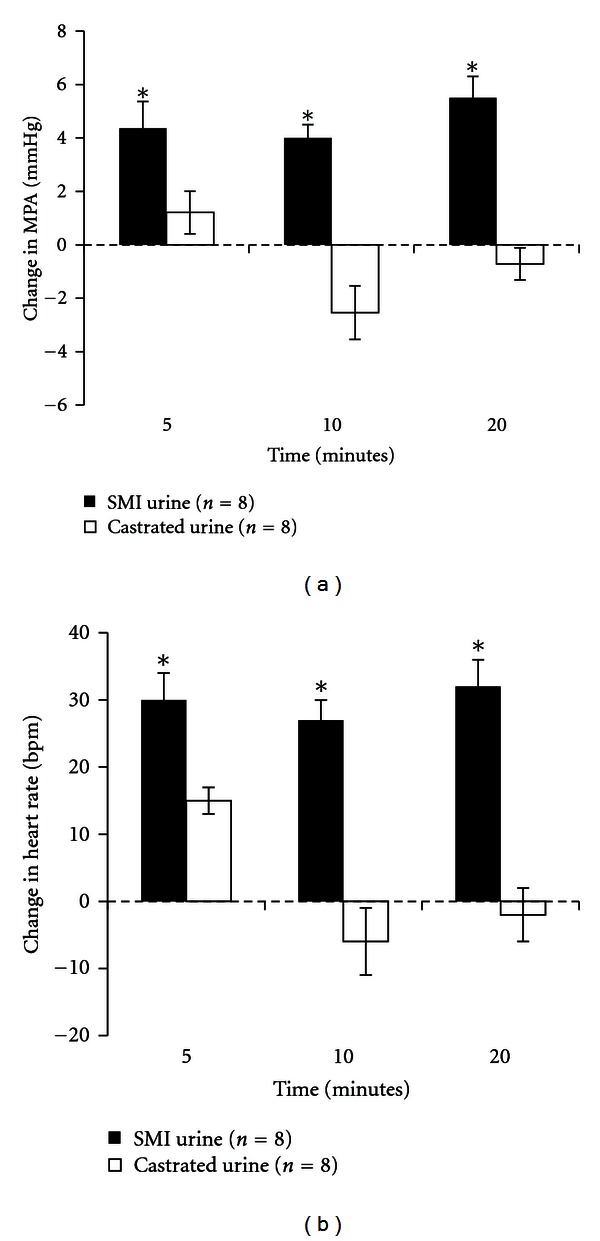
The changes in the hypertensive (a) and heart rate (b) responses of restrained male mice during the 5-, 10- and 20-minute marks due to the exposure of urine from SMI male mice or urine from castrated mice. *Indicates a significant difference in the responses (*P* < 0.05).

Data are expressed as a change from baseline values with means ± SE. The mean arterial pressure (MAP), heart rate (HR), and locomotor activity data were collected from 3 PM until 10 AM (i.e., 19 hours) every day. The 3 PM–6 PM period and the 6 AM to 10 AM period together were analyzed as “day” MAP, and the 6 PM to 6 AM period (12 h) was “night.” On the day of the experiment, the average baseline values were taken from the previous two hours before introducing urine from SMI or castrated males or saline. MAP, HR, and motor activity data were collected at 500 Hz for 5 s/min throughout the baseline and stress periods and then were averaged in 5-minute intervals. MAP, HR, and motor activity are expressed as millimeters of Hg, beats per minute, and counts, respectively. Each point on the graphs represents an average of five 1-minute data points for MAP, HR, and activity. Data in Figures 1(a)–1(c)represent a total of 34 mice. Data in Figures 2(a) and 2(b) represent a total of 16 mice.

### 2.2. Data Analysis

 The average baseline values (MAP, HR, locomotor activity) were taken from the last 120 min before the free-roaming experiments. The average baseline values (MAP and HR) were taken 25–30 minutes before a solution was introduced to a mouse in the restrainer. All data were analyzed by SPSS (Chicago, IL) computer software. Data were analyzed with repeated-measures ANOVA to take into account the magnitude and duration of the effects. Significant F-tests from the ANOVA at *P* < 0.05 were followed by post hoc comparisons using the Newman-Keuls multiple range test. Statistical significance was set at *P* < 0.05. The statistical comparisons were made across the multiple groups. The normality of data distribution was checked by utilizing the Shapiro-Wilks of Kolmogoron-Smirnov test in the SPSS software.

### 2.3. Urine Collections

 Sexually mature intact male mice (10 weeks old) were placed in individual metabolic cages (Braintree) that minimized food and fecal contamination of urine samples for 24 hours. Twenty-four hour periods were used to collect urine and monitor food and water intake. Food and water were available ad libitum. A separate set of sexually mature male mice were castrated and allowed to recover for 14 days prior to their placement in individual metabolic cages. Urine samples were collected and stored at −80°C until the day of the experiment.

## 3. Results

**Free-Roaming Conditions**

Baseline mean arterial pressure (MAP), heart rate (HR), and locomotor activity were collected two hours prior to the placement of either SMI or castrated urine or saline in the cage bedding. Baseline measurements for MAP, HR, and locomotor activity were 99 ± 5 mmHg, 527 ± 13 bpm, and 5 ± 1, respectively (Table 1). Urine from SMI male mice caused a significant peak change in MAP (21 ± 4 mmHg) that occurred five minutes after placing the urine in the cage-bedding. The change in MAP (4 ± 3 mmHg), 35 minutes after SMI urine exposure, did not return to the average baseline value (Figure 1(a)). Urine from SMI male mice caused a significant peak change in heart rate (145 ± 25 bpm) that occurred five minutes into the experiment. The change in heart rate (45 ± 20 bpm) also did not return to baseline values after approximately 35 minutes (Figure 1(b)). Urine from SMI male mice elicited a peak change in locomotor activity (9 ± 2 counts) that occurred five minutes into the experiment (Figure 1(c)). Saline did not elicit a significant change in MAP, HR, or locomotor activity (Figures 1(a)–1(c)).

Urine from castrated male mice elicited a peak change MAP (11 ± 3 mmHg) that occurred 10 minutes after placing the urine in the cage-bedding. The change in the MAP response to castrated male urine returned to baseline at the 35-minute mark (Figure 1(a)). Urine from castrated male mice elicited a peak change in heart rate of 83 ± 15 bpm that occurred ten minutes into the experiment (Figure 1(b)). The change in heart rate (10 ± 7 bpm) did not completely return to baseline values after 35 minutes. The peak change in locomotor activity was  5 ± 1  counts and occurred during the first five minutes of the experiment. The change in locomotor activity during the castrated urine application returned to baseline values after 35 minutes (Figure 1(c)).

 Terazosin significantly reduced baseline MAP and locomotor activity, while increasing HR. Baseline MAP, HR, and locomotor activity averaged 91 ± 5 mmHg, 595 bpm, and 1 count, respectively. Terazosin attenuated the peak change in MAP (9 ± 3 mmHg), HR (90 ± 15 bpm), and motor activity  4 ± 2  counts compared with SMI urine-induced responses (Figures 1(a)–1(c)).

 The analysis involved comparisons across multiple groups. Urine from SMI mice caused significant increases in the magnitude of the MAP, HR, and locomotor activity responses when compared to castrated urine, SMI urine-terazosin treatment, and saline applications. The magnitude of the MAP, HR, and locomotor activity responses to castrated urine and SMI urine-terazosin treatment were also significantly higher than those elicited by saline. The MAP, HR, and locomotor activity responses during SMI urine-terazosin treatment were not significantly different from responses elicited by urine from castrated mice. In addition, saline did not elicit significant increases in MAP, HR, and locomotor activity responses of the Terazosin-treated mice (data not shown).

**Restrainer Studies**

In the restrainer study, SMI urine induced a change in mean arterial pressure of 4.4 ± 1, 4.0 ± 0.5, and 5.5 ± 1 mmHg at minutes 5, 10, and 20, respectively (Figure 2(a)). The change in HR during the SMI urine experiment was 30 ± 4,  27 ± 3, and 32 ± 4 bpm at minutes 5, 10, and 20, respectively (Figure 2(b)). Urine from castrated mice caused a modest increase in the change of mean arterial pressure (1.2 ± 0.8 mmHg) after 5 minutes. During minutes 10 and 20, the change in mean arterial pressure fell below baseline values. The peak change in HR during the application of urine from castrated mice was 15 ± 2 bpm. Changes in heart rate returned to baseline values prior to the 10-minute mark. The SMI urine-induced MAP and HR responses were significantly higher than the castrated urine-induced MAP and HR responses.

The change in MAP and HR responses during the application of saline did not differ significantly from the change in MAP and HR responses to urine from castrated mice. Saline elicited a peak change in MAP (1.8 ± 0.3 mmHg) and HR (6.1 ± 3 bpm). The MAP and HR responses in mice pretreated with terazosin and exposed to SMI urine were not significantly different from responses elicited by castrated urine and saline (data not shown).

## 4. Discussion

The main findings from this study are the following.

1. During free-roaming conditions, pheromones in the urine of sexually mature intact male mice contributed to significant increases in mean arterial pressure, heart rate, and locomotor activity when placed in the cage bedding of an individually housed free-roaming male mouse.
2. The rapid peak increase in MAP following the application of urine from sexually mature intake male mice is due, to a significant extent, to *α*_1_-adrenergic receptor activation.
3. Urine from castrated mice elicited MAP, HR, and locomotor activity responses that were significantly lower than responses induced by SMI male mice.
4. During restraint, SMI urine induced a significant increase in MAP and HR responses when compared to MAP and HR responses induced by urine from castrated mice.
5. Saline administration during restraint did not induce a significant increase in MAP and only a transient increase in heart rate.

Mouse social culture is not commonly controlled for by researchers and housing animals in different groups upon arrival has been shown to alter social structure, stress the mice, and contribute to elevated blood pressure [7–9]. Within the mouse social culture, pheromones regulate many aspects of mouse interactions, including individual recognition, reproduction cycles, gender identification, and aggression between male mice [10]. A recent study demonstrates that mice use cues from pheromones to determine if defense mechanisms or protection of resources are needed [6]. Although the detection of pheromones is known to promote aggression between male mice, the findings from the current study suggest that the detection of sexually mature intact urine pheromones by an individually housed male mouse or a restrained male mouse contributes to significant increases in mean arterial pressure and heart rate responses.

A previous study determined that at least two chemically distinct ligands are sufficient to promote male-male aggression and stimulate vomeronasal organ neurons [6]. The specific aggression-promoting activity is dependent on the presence of the protein component of the major urinary protein complex, which is comprised of specialized lipocalin proteins bound to small organic molecules [11, 12]. Synthetic pheromones, 2-sec-butyl dihydrothiazole and dehydro-exo-brevicomin, have been shown to act synergistically and promote intermale aggression in mice when added to castrated male urine but not when added to water. Therefore, it should not be totally surprising that urine from castrated mice caused a hypertensive response in the current study. Castration has been shown to reduce urinary volatile compounds; however behavior tests with these compounds are necessary to show the possible chemo-signaling and physiological functions [13]. Castration also significantly reduces the production of lactones, which may be mediators of chemical communication [13]. The synthetic pheromones provoke fighting that is quantitatively and qualitatively comparable to that elicited by intact male urine [3]. Results from this study and previous studies suggest that male mice use male urinary chemicals as fundamental cues to the presence of an adult opponent male. In such cases of competitive aggressive behavior, these signals can mislead the resident male to prepare for preventive attacks [1, 14]. Future studies are needed to identify the specific pheromones in sexually mature intact male urine that are responsible for inducing increases in mean arterial pressure, heart rate, and locomotor activity.

The regulation of blood pressure is through a complex interaction of neural, hormonal, vascular, cardiac, and renal mechanisms. Mouse models of hypertension have been used to explain the complex interactions that exist during the initial stages and maintenance of hypertension. Many researchers involved in hypertension research routinely measure blood pressure in male mice via telemetry or tail cuff [15]. During the studies, it is common for male mice to be placed in cages previously occupied by different male mice or placed in a restrainer previously occupied by a male mouse. Tail cuff has been known to overestimate stress-induced hypertension compared to direct blood pressure measurements [16]. Despite the well-intended efforts by researchers to train and acclimatize animals to undergo the tail cuff measurements, these methods impose significant stress and disturbs multiple aspects of the cardiovascular system [15]. The acclimatization process to tail-cuff procedures still leaves doubt about the impact of the procedural stress on cardiovascular function [17]. The present study suggests that pheromones in the urine of SMI male mice may elevate mean arterial pressure and heart rate during tail-cuff measurements.

 In summary, our studies suggest that pheromones in the urine of sexually mature intact male mice contribute to significant increases in mean arterial pressure, heart rate, and locomotor activity. During blood pressure measurements, researchers should account and control for the contribution of male urinary pheromones to their data. Although researchers are less likely to move male mice from cage to cage while using biotelemetry, the detection of pheromones in the urine of male mice will significantly contribute to acute changes in blood pressure, heart rate, and locomotor activity. However, special attention and care should be given to the cleaning of mouse restrainers during tail-cuff measurements that involve male mice. Although the amount of urine that male mice are exposed to varies, future studies are also needed to determine if an accumulation of SMI urine pheromones elicits a further increase in mean arterial pressure, heart rate, and locomotor activity responses. It is also not known if repeated exposures to urinary pheromones from sexually mature intact males cause a sustained increase of the cardiovascular responses.

## Figures and Tables

**Table 1:** Baseline measurements.

Condition	MAP	Heart Rate	Locomotor activity
Free roaming	99 ± 5 mmHg	527 ± 13 bpm	5 ± 1 counts
Restrainer	121 ± 4 mmHg*	745 ± 4 bpm*	—

Baseline measurements of mean arterial pressure (MAP), heart rate, and locomotor activity of mice two hours prior to being tested in the free-roaming experiment and twenty-five minutes prior to the restraint experiments. *Indicates a significant difference between the respective groups (*P* < 0.05).
